# The impact of user fees on health services utilization and infectious disease diagnoses in Neno District, Malawi: a longitudinal, quasi-experimental study

**DOI:** 10.1186/s12913-016-1856-x

**Published:** 2016-10-20

**Authors:** S. I. Watson, E. B. Wroe, E. L. Dunbar, J. Mukherjee, S. B. Squire, L. Nazimera, L. Dullie, R. J. Lilford

**Affiliations:** 1 University of Warwick, Coventry, UK; 2 Partners In Health/Abwenzi Pa Za Umoyo, PO Box 56, Neno, Malawi; 3 Brigham & Women’s Hospital, Boston, USA; 4 Partners In Health, Boston, MA USA; 5 Liverpool School of Tropical Medicine, Liverpool, UK; 6 Ministry of Health, Neno District, Malawi

**Keywords:** User fees, Healthcare utilisation, Malaria, HIV, Tuberculosis, Access to health care, Universal health coverage

## Abstract

**Background:**

User fees have generally fallen out of favor across Africa, and they have been associated with reductions in access to healthcare. We examined the effects of the introduction and removal of user fees on outpatient attendances and new diagnoses of HIV, malaria, and tuberculosis in Neno District, Malawi where user fees were re-instated at three of 13 health centres in 2013 and subsequently removed at one of these in 2015.

**Methods:**

We conducted two analyses. Firstly, an unadjusted comparison of outpatient visits and new diagnoses over three periods between July 2012 and October 2015: during the period with no user fees, at the re-introduction of user fees at four centres, and after the removal of user fees at one centre. Secondly, we estimated a linear model of the effect of user fees on the outcome of interest that controlled for unobserved health centre effects, monthly effects, and a linear time trend.

**Results:**

The introduction of user fees was associated with a change in total attendances of −68 % [95 % CI: −89 %, −12 %], similar reductions were observed for new malaria and HIV diagnoses. The removal of user fees was associated with an increase in total attendances of 352 % [213 %, 554 %] with similar increases for malaria diagnoses. The results were not sensitive to control group or model specification.

**Conclusions:**

User fees for outpatient healthcare services present a barrier to patients accessing healthcare and reduce detection of serious infectious diseases.

**Electronic supplementary material:**

The online version of this article (doi:10.1186/s12913-016-1856-x) contains supplementary material, which is available to authorized users.

## Background

The promotion of user fees as a finance mechanism for health services in low and middle income countries (LMICs) is generally no longer favoured [1, 2], despite the 1987 Bamako Initiative promoted by the WHO and UNICEF, which recommended user fees to improve healthcare quality. In recent years, several African nations have eliminated fees [3, 4]. In an analysis of 56 intergovernmental and international non-governmental organizations, government agencies, and other networks, no global health actor was in support of user fees or against free care at the point of service. However, there is no clear consensus on the appropriate action, and some actors – more commonly government agencies – remain silent [1]. User fees may impose a barrier to care and thus threaten progress toward universal health coverage, and no nation has achieved universal health coverage through a system based on out-of-pocket payments [3, 5, 6].

There have been a number of studies examining the consequences of introducing or removing user fees, particularly in sub-Saharan Africa [7]. These generally suggest increased utilization with the abolition of user fees, or conversely a reduction in utilization with their introduction, although the quality of the evidence has been questioned [7–12]. For example, studies indicate an increase in facility based deliveries across several African countries with the removal of user fees [4, 13]. One also indicated a possible improvement in neonatal mortality, examining routine data from Kenya, Senegal, and Ghana, with seven other African nations as controls [4]. A modelling study also predicted that removing user fees in 20 African countries would significantly reduce under five mortality, assuming that poor people would be the main beneficiaries [14]. Many studies indicate that user fees have a greater impact in poor populations, although there is general agreement that in addition to user fees there are other barriers for the poor that need addressing [4, 13, 15].

Randomised trials of the introduction or removal of user fees in LMICs are rare. Two studies, one in Ghana [16] and one in Afghanistan [17], both showed large increases in the use of healthcare after the removal of fees in randomised trials. The former trial, which examined children under 5, found evidence of increased formal primary care utilisation, but not for a subsequent effect clinical outcomes of malaria. The latter trial found the removal of user fees led to a 400 % increase in the utilisation of health services.

Malawi has provided free public health care since September 1964, resisting international pressures to introduce fees at several points since its independence. However, 24 % of health centres are operated by the Christian Health Association of Malawi (CHAM), an umbrella-group of 169 facilities that are independently operated by church-affiliated, not-for-profit groups. The MOH negotiates Service Level Agreements (SLAs) with CHAM at the district-level; thus across Malawi there is a patchwork of free health services while the majority of CHAM facilities charge user fees [18].

In Neno District, there has been a pattern of free and fee-for-service at outpatient departments based on SLAs established between CHAM facilities and the Ministry of Health. In this study we examined the effects of this natural experiment on health care attendances using a Ministry of Health database of routine care at all 13 facilities in the district. This helps inform what happens to patient volume in rural Malawi when user fees are removed and, if expanded, could help inform operational planning. We also used the routine health data set to explore the effects on new diagnoses of malaria, tuberculosis, and HIV. This study presents important new evidence on the effects and possible health consequences of user fees in a low-income country at a time when many governments are focusing both on implementation of universal health coverage and increasing HIV case finding to reach the UNAIDS 90-90-90 goals. This study also investigates the relationship of user fees and HIV case detection.

## Methods

### Study background and context

In Neno District, Malawi the health care infrastructure consists of 13 different healthcare facilities: eight are operated by the Ministry of Health, one private facility operated by a local electric company, and four are administered by CHAM. In recent years, there have been several shifts in the implementation of user fees at CHAM facilities across the district.

Partners In Health (PIH), an NGO known in Malawi by its Chichewa name Abwenzi Pa Za Umoyo, works with MOH to strengthen health systems and helped broker SLAs with the CHAM facilities (Matope, Matandani, and Nsambe). In July 2013, these three CHAM facilities terminated their Service Level Agreements and introduced user fees simultaneously for general outpatient visits. It was replaced with an SLA covering free maternal, neonatal, and HIV services, meaning user fees were instituted for all other outpatient visits. User fees comprise consultation fees for seeing a clinician, fees for laboratory tests, and fees for medications. This re-institution of user fees has been seen across Malawi, as previous funding for SLAs was withdrawn by health donors, prompting re-initiation of user fees at many CHAM facilities across the country. In 2016, the government of Malawi is pursuing an agreement with CHAM that will allow districts to independently proceed once again with these SLAs; however, funding limitations remain a significant barrier in most districts [19].

**Fig. 1:**
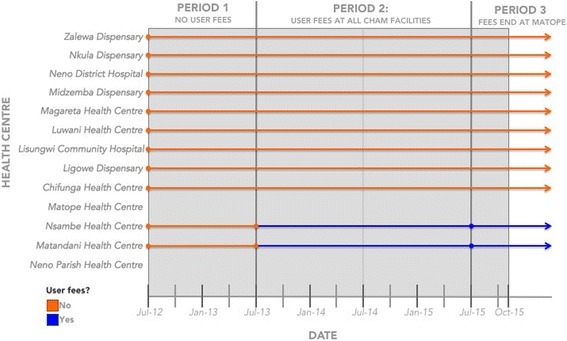
Implementation of user fees across health centres in Neno District, Malawi

**Fig. 2:**
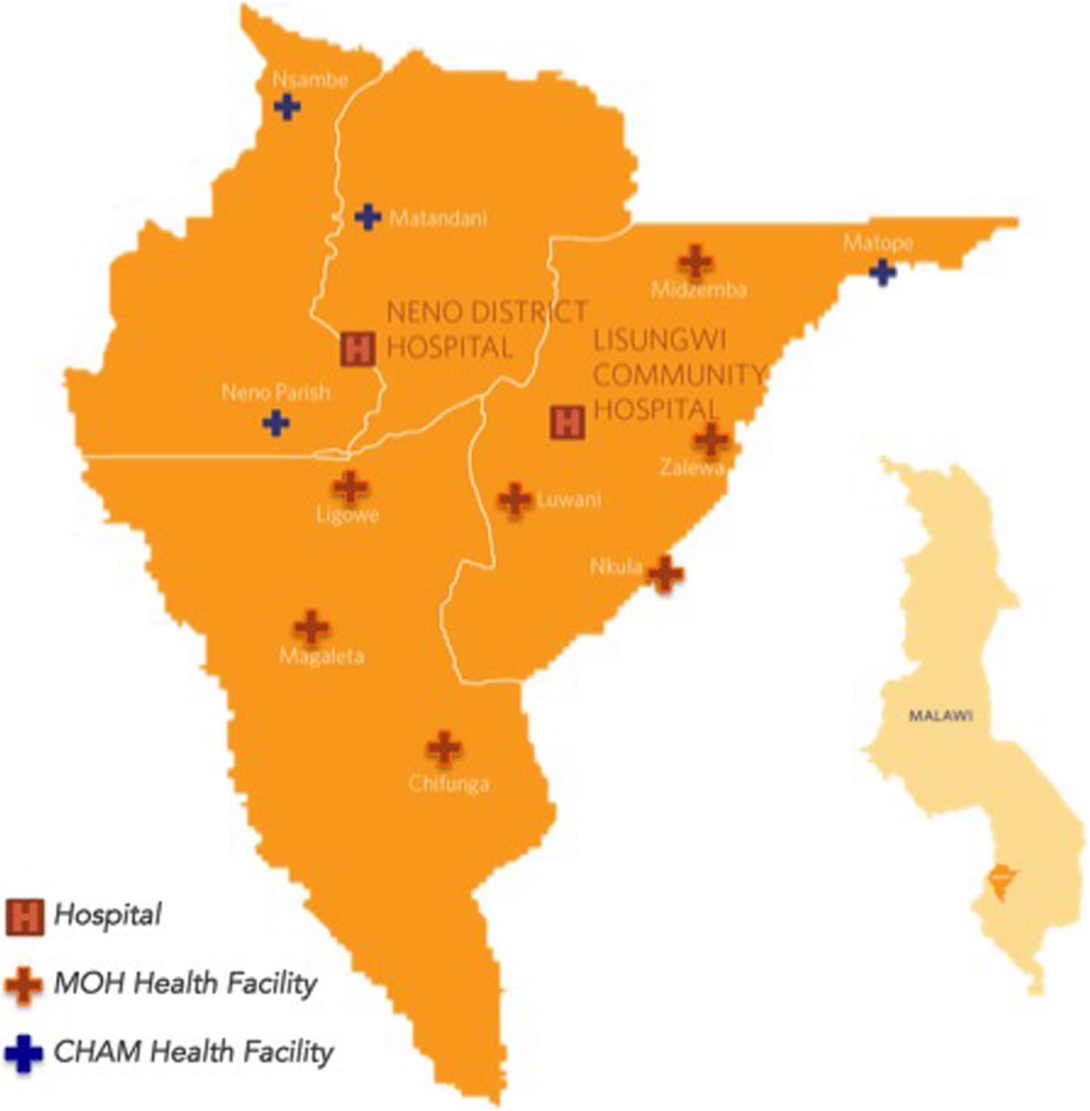
Map of Neno District, Malawi and location of healthcare centres

Because the assistance of PIH is available in Neno District, in July 2015, user fees were eliminated at one of these three centres (Matope) through a new SLA. One centre (Neno Parish) charged user fees for the duration of the study period. The remaining nine facilities did not charge user fees. Figure 1 shows the periods when each centre did or did not charge user fees. Figure 2 shows the location of each of the centres.

### Statistical analysis

The aim of the study was to identify the effects of introducing or removing user fees on attendances and diagnoses of communicable diseases at health centres in Neno District, Malawi. In particular, the outcomes analysed were: total outpatient attendances, total number of new malaria diagnoses in patients aged under 5, total number of new malaria diagnoses in patients aged over 5, and total number of new confirmed HIV cases in patients aged 15 to 49. HIV care, once diagnosed, was free to patients throughout the entire time period. New HIV diagnoses were examined because of the opportunity for HIV case-finding during outpatient visits for acutely ill clients. Data were available on the number of new TB diagnoses, however this was not included in the regression analyses as the outcome was rare and could not be analysed.

The use of routinely collected longitudinal data enabled us to take into account effects that may have confounded our analyses including secular trends in health care utilisation, seasonal effects, and unobserved health centre effects. The analysis presented here can be considered a generalisation of a difference in differences (DiD) regression model with multiple units in the treatment (user fee charging) and control (no user fees) groups and multiple time periods.

We specified a linear model. The dependent variable was the natural logarithm of the number of health care attendances or new diagnoses of the nominated diseases. We included in the model different intercepts for each health care facility, monthly dummy variables, and a treatment group (user fees or no user fees) dummy variable. We also included a linear time trend interacted with the treatment group dummy variable: this allows for “correlated random trends”, which relaxes the parallel trends assumption normally required for DiD [20]. The user fee and non-user fee groups may have different trends over time in health services utilisation and these trends may be correlated with the introduction or removal of user fees. For example, user fees may have been introduced in response to declining attendances. We considered that the introduction and removal of user fees would have differential treatment effects. We therefore estimated the effects of the introduction and removal of user fees separately. The standard errors were clustered at the health centre level.

The primary analysis may under or overestimate the effect of introducing user fees, since many users may travel to a different centre that does not charge a user fee. These individuals may not choose to attend a health centre had there been user fees implemented at all centres. As a sensitivity analysis we considered a different control group: the subset of non-user fee charging facilities separated from a user fee charging facility by another non-user fee charging facility (Magaleta, Chifunga, Luwani, and Nkula). We considered using a prior, formal rule to categorise centres in this regard, but chose simple discrimination based on visual inspection of the location of centres (see Fig. 2) since we discerned no ambiguous cases. We also considered different model specifications: a fixed-effects model that does not allow for “correlated random trends”, and a fixed effects Poisson regression. Finally, we excluded Nkula from the analyses since it was not operated by MOH or CHAM.

### Data and sample selection

Routinely collected data from the “HMIS-15” report were extracted from Malawi’s District Health Information Software 2 (DHIS2) for this analysis. The “HMIS-15” report summarizes core health service utilization at each facility including maternal health, antenatal care, HIV diagnoses, and outpatient department visits. No formal data quality assessments on the HMIS-15 report were performed during the study period to assess the accuracy and validity of these data; however spot checks on major outliers were conducted. The period for the analyses was July 2012 to October 2015.

### Ethical considerations

Ethics committee approval was obtained for analysis and publication of routinely collected data to evaluate services within Neno District from both the Malawi National Health Sciences Research Committee (Lilongwe, Malawi) and Partners Institutional Review Board (Boston, MA). Aggregated datasets were utilized, thus individual informed consent was not obtained.

## Results

### Summary statistics

**Fig. 3:**
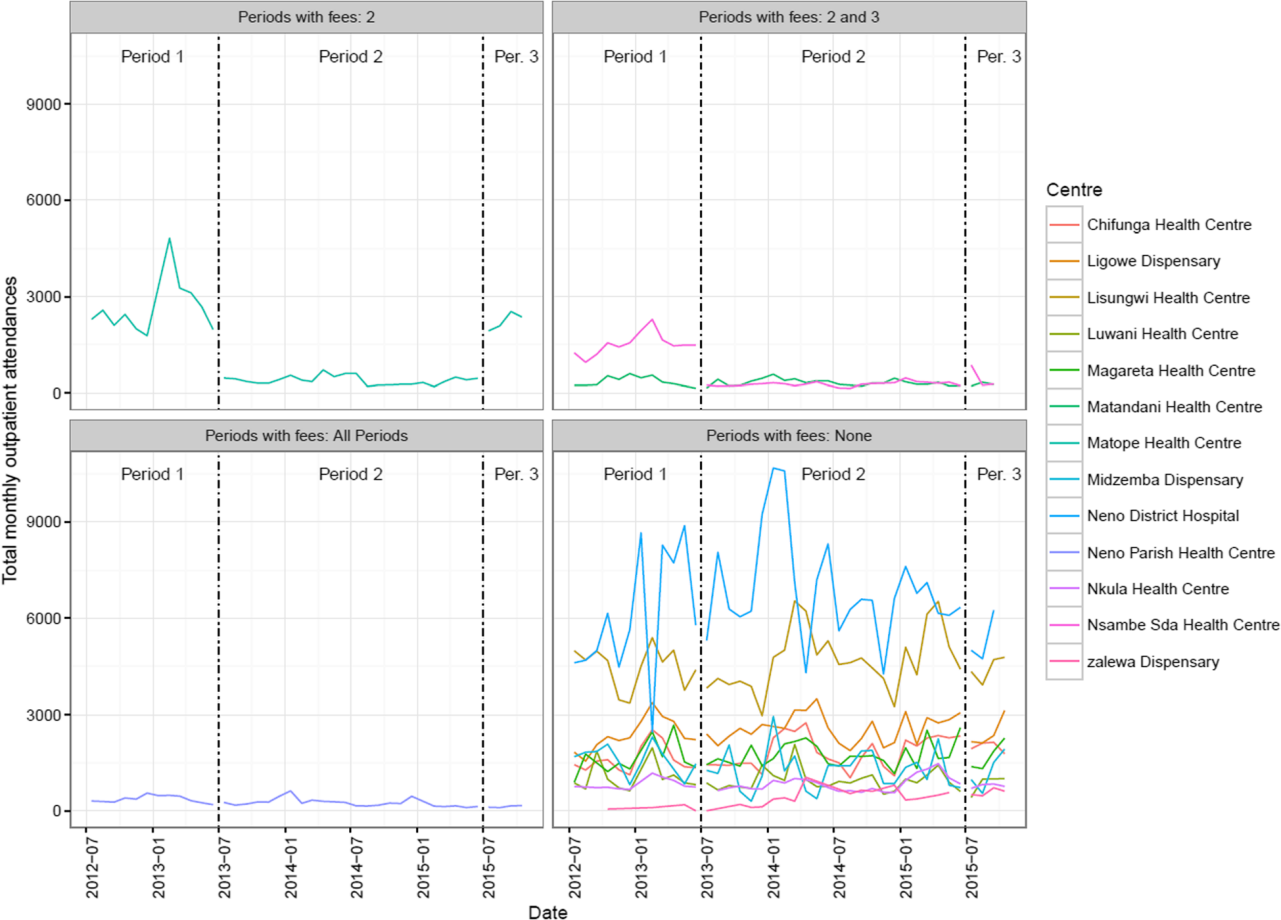
Monthly number of total outpatient attendances at health centres in Neno District, Malawi for the period July 2012 to October 2015 by user fee status

Table 1 shows summary statistics of the data by period: period one is July 2012 to June 2013, period two is July 2013 to June 2015, and period three is July 2015 to October 2015. These periods correspond to changes in the health centres that charged patients user fees (Fig. 1). Following the introduction of user fees in period two, average monthly outpatient attendances were 15 %, 18 %, and 90 % of pre-user fee levels at Matope, Nsambe, and Matandani, respectively. Attendances remained at 97 % of the pre-user fee level, on average, at Ministry of Health centres over the same two periods. After the removal of user fees in period three, average monthly attendances at Matope returned to 83 % of their pre-user fee level. Figure 3 shows the monthly attendances at all centres in Neno District, Malawi with trends by period.

**Table 1:** Average monthly numbers of patients at centres by whether user fees were charged

	User fees group (periods in which fees were charged)	Period 1: Before user fees at CHAM (July 2012 to June 2013)	Period 2: User fees at all CHAM centres (July 2013 to June 2015)	Period 3: Removal of user fees at Matope (July 2015 to October 2015)
Total outpatient attendances	MOH Facilities without fees	21,818 (9,629) [100]	21,193 (3,089) [97]	18,611 (2,409) [85]
	CHAM Facilities with Fees in Period 2 and 3	1,875 (453) [100]	602 (133) [32]	726 (298) [39]
	CHAM Facility with Fees in Period 2	2,695 (845) [100]	391 (135) [15]	2,227 (271) [83]
	CHAM Facility with Fees in All periods	364 (110) [100]	252 (121) [69]	133 (31) [37]
	All Centres	26,752 (10,266) [100]	22,624 (3,170) [85]	21,642 (2,598) [81]
Total new malaria diagnoses, under 5 s	MOH Facilities without fees	2,744 (588) [100]	2,317 (780) [84]	1,806 (225) [66]
	CHAM Facilities with Fees in Period 2 and 3	331 (272) [100]	257 (117) [78]	385 (341) [116]
	CHAM Facility with Fees in Period 2	305 (194) [100]	63 (27) [21]	183 (44) [60]
	CHAM Facility with Fees in All periods	123 (60) [100]	83 (57) [67]	21 (16) [17]
	All Centres	3,558 (815) [100]	2,753 (899) [77]	2,395 (272) [67]
Total new malaria diagnoses, over 5 s	MOH Facilities without fees	4,397 (1,551) [100]	4,193 (1,718) [95]	2,938 (294) [67]
	CHAM Facilities with Fees in Period 2 and 3	435 (301) [100]	199 (139) [46]	319 (159) [73]
	CHAM Facility with Fees in Period 2	467 (57) [100]	105 (57) [22]	373 (112) [80]
	CHAM Facility with Fees in All periods	112 (21) [100]	84 (57) [75]	21 (26) [19]
	All Centres	5,569 (1,550) [100]	4,680 (1,891) [84]	3,627 (413) [65]
Total new TB diagnoses	MOH Facilities without fees	13 (4) [100]	9 (4) [69]	12 (6) [92]
	CHAM Facilities with Fees in Period 2 and 3	0 (0) [−]	0 (0) [−]	1 (2) [−]
	CHAM Facility with Fees in Period 2	1 (1) [100]	0 (1) [0]	1 (1) [100]
	CHAM Facility with Fees in All periods	0 (1) [−]	0 (1) [−]	0 (0) [−]
	All Centres	15 (5) [100]	9 (3) [60]	14 (5) [93]
New confirmed HIV+ patient, aged 15-49	MOH Facilities without fees	95 (43) [100]	100 (92) [105]	89 (23) [94]
	CHAM Facilities with Fees in Period 2 and 3	20 (33) [100]	7 (4) [35]	9 (4) [45]
	CHAM Facility with Fees in Period 2	15 (10) [100]	6 (4) [40]	7 (6) [47]
	CHAM Facility with Fees in All periods	2 (1) [100]	1 (1) [50]	1 (2) [50]
	All Centres	129 (59) [100]	114 (95) [88]	114 (14) [88]

Figures are mean (sd) [as % of period 1 values]. The groups of centres are: *no user fees* were charged in nine ministry of health centres Chifunga, Ligowe, Lisungwi, Luwani, Magareta, Midzemba, Neno District Hospital, Nkula, and Zalewa; user fees were charged in *Periods 2 and 3* in two CHAM centres Nsambe and Matandani; user fees were charged only in *Period 2* in one CHAM centre Matope; and one CHAM centre charged user fees in *All Periods:* Neno Parish

The average monthly numbers of new diagnoses of malaria in both the under 5 s and over 5 s as well as new confirmed HIV cases also decreased in period 2 for centres that introduced user fees: to 50 % of the pre-user fee level for malaria diagnoses in the under 5 s, 33 % for malaria diagnoses in the over 5 s, and 37 % for new confirmed HIV cases. For tuberculosis, the number of new diagnoses per month was small; however, the total number of new diagnoses of TB in the year following the introduction of user fees at user fee introducing centres was 9 compared to 16 the year before. Similar declines were not seen in the Ministry of Health centres that did not introduce user fees.

### Main results

The results from the main analyses are presented in table 2. Overall, there was good evidence to confirm what was observed in the raw data: the presence of user fees changed total attendances by −68 % [95 % confidence interval: −89 %, −12 %], new malaria diagnoses in the over 5 s by −56 % [−83 %, +14 %], and new confirmed HIV cases in people aged 15–49 by −48 % [−64 %, −25 %]. The estimated change in new malaria diagnoses among under 5 s were also negative but accompanied by wide confidence intervals.

**Table 2:** Main results: estimated effect of the presence of user fees on monthly attendances and diagnoses at health centres in Neno District, Malawi

	Total outpatient attendances (subject to user fees)	Total new malaria diagnoses, under 5 s	Total new malaria diagnoses, over 5 s	New confirmed HIV+ patient, aged 15–49
Periods 1 and 2: Introduction of user fees^a^				
Estimated change (%)	−68	−18	−56	−48
95 % confidence interval	[−89, −12]	[−73, 144]	[−83, 14]	[−64, −25]
*P*-value	0 · 048	0 · 70	0 · 084	0 · 002
Number of centres	13	13	13	13
Number of months	35	35	35	35
Periods 2 and 3: Removal of user fees^b^				
Estimated change (%)	352	230	247	-^c^
95 % confidence interval	[213, 554]	[106, 430]	[171, 343]	-
*P*-value	<0 · 001	<0 · 001	<0 · 001	-
Number of centres	13	13	13	13
Number of months	27	27	27	27

The estimated change represents the average difference in monthly attendances or diagnoses associated with the introduction or removal of user fees

^a^Estimated using data from the period July 2012 to June 2015

^b^Estimated using data from the period August 2013 to October 2015

^c^There were two few new confirmed HIV+ patients treated at the centre which removed user fees to analyse

The effect of removing user fees was to significantly increase attendances at the health centres. In particular, the removal of user fees resulted in an increase in total outpatient attendances of 352 % [213 %, 554 %], largely returning the number of attendances to their pre-user fee level. Similar increases were seen in the numbers of new malaria diagnoses in the under 5 s and over 5 of 230 % [106 %, 430 %] and 247 % [171 %, 343 %], respectively. There were too few new confirmed HIV cases in the centre that removed user fees to analyse.

### Robustness and sensitivity analyses

We conducted a sensitivity analysis by restricting the control group, i.e. MOH centres that offered free services throughout study period and were geographically separated from a user-fee-introducing or -removing centre by at least one other non-user fee centre to account for patient potentially travelling to other centres. These results are presented in Table 3. The results are qualitatively, highly similar to the main results presented in Table 2, although the confidence intervals are widened by the reduction in power.

**Table 3:** Results from sensitivity analyses which restricted the control group to MOH health centres offering free services through study period and geographically separated from CHAM user fee introducing/removing centres

	Total outpatient attendances	Total new malaria diagnoses, under 5 s	Total new malaria diagnoses, over 5 s	New confirmed HIV+ patient, aged 15–49
Periods 1 and 2: Introduction of user fees^a^				
Estimated change (%)	−70	−22	−56	−52
95 % confidence interval	[−92, 8]	[−78, 177]	[−86, 40]	[−77, 1]
*P*-value	0 · 060	0 · 66	0 · 14	0 · 051
Number of centres	8	8	8	8
Number of months	35	35	35	35
Periods 2 and 3: Removal of user fees^b^				
Estimated change (%)	345	170	261	-^c^
95 % confidence interval	[136, 737]	[13, 545]	[130, 467]	-
*P*-value	0 · 001	0 · 031	<0 · 001	-
Number of centres	8	8	8	8
Number of months	27	27	27	27

The estimated change represents the average difference in monthly attendances or diagnoses associated with the introduction or removal of user fees. Standard errors were clustered at the health centre level

^a^Estimated using data from the period July 2012 to June 2015

^b^Estimated using data from the period August 2013 to October 2015

^c^There were two few new confirmed HIV+ patients treated at the centre which removed user fees to analyse

We also tested the sensitivity of results to regression model specification. These results are presented in Tables A1 and A2, Additional file 1. Results from both model specifications were qualitatively very similar to the base case results presented in Table 2. Results were also robust to the removal of one centre administered by neither the MOH or CHAM (Nkula).

## Discussion

This study has provided estimates of the effect of introducing and removing user fees for outpatient utilization services in Neno District, Malawi. We showed that the introduction of user fees led to large, significant declines in outpatient attendances, which also translated into an indirect effect of reductions in new diagnoses of malaria and HIV. The removal of user fees largely reversed this effect. We also showed that the results were very similar when the control group was restricted to health centres that were geographically separated from centres that introduced or removed user fees, which suggests patients were not travelling to other health centres for care. This was also reflected by a lack of an increase in outpatient attendances at the main district hospital.

Our results on overall health-facility attendances support those from other similar studies [7], but also reveal the indirect impact on identifying new cases of infectious diseases such as malaria and HIV. This study in conjunction with previous analyses suggests user fees may have a pernicious effect on public health. Despite free HIV services provided throughout, it could be that the opportunity to test patients during other sick visits contributed to the reduced new HIV cases diagnosed during periods charging user fees. This is supported because the vast majority of HIV testing in Malawi is among outpatients, as well as the absence of significant changes in HIV funding, implementing partners, or testing guidelines throughout the study period. Our results do not support the idea that user fees select for the urgent cases where access to healthcare is the most critical; user fees would appear to be largely non-discriminatory.

We did not study other outcomes, but other critical conditions in Neno that may be diagnosed at outpatient clinics include paediatric malnutrition, diarrheal disease and other infections. Since outpatient department patient visits declined with implementation of user fees and consequently new diagnoses decrease, many sick patients with important chronic illnesses will remain undetected in the community as overall case detection rates declines. Over the 24-month period from July 2013 to July 2015, the four, user-fee-charging facilities had a combined average of 5,340 outpatient attendances per 10,000 population versus 51,820 visits per 10,000 population in the free facilities; a WHO Health Systems Strengthening indicator that can indicate poor availability and quality of services [21]. This represents an estimated loss of 270,000 sick-patient visits over the two year period user fee areas. As a reflection of this, in January 2015, a free, mobile clinic in response to emergency flooding in Matope, clinicians treated 662 patients in a single day, a third of whom had a positive malaria rapid diagnostic test highlighting the community’s urgent need for access to healthcare (unpublished data).

The long-term health consequences of user fees are uncertain. Health outcomes in low-income countries are not well reported, especially for people not in contact with the healthcare system. Nevertheless, given the low outpatient department attendance at user fee facilities, it is possible that patients tend to seek care later in the course of their disease, leading to, for example, more weight loss and complications in HIV and TB patients, and a longer recovery period. Ultimately, delays in diagnosis and treatment increase the risk of costly hospitalization, disease transmission and death [22–24]. Removing user fees in settings like Neno District, which serve amongst the poorest people globally, may therefore be an effective way of reducing the impact of serious communicable diseases. Malaria, HIV, and tuberculosis account for 5.6 million deaths and the loss of 166 million disability adjusted life years annually [25].

User fees have also previously been shown to contribute to the sometimes catastrophic cost burden of illness among the poor in low income countries and prevent access to preventative or curative services [26]. In Malawi, where the average income is 84 cents per day [27], the costs faced by patients are prohibitive. In Neno District, a patient might expect to walk for 30 min to 2 h to reach the nearest health centre; if avoiding a facility with user fees, this travel time may increase to up to 5 to 10 h (assuming a 3 · 5 km/h walking time) [28]. A bicycle taxi is unaffordable for most, with a round trip costing approximately 500 to 1000 MWK (1–2 USD), nearly 3-days pay. If the patient is seen for an upper respiratory tract infection or diarrheal illness a patient might expect to pay 300 to 500 MWK (0 · 6-1 USD) for the consultation and another 800 to 1000 MWK (1 · 6-2 USD) for the prescribed medications (unpublished data).

We acknowledge weaknesses to our study. This study was done in one district in Malawi. Additionally, while we have attempted to identify the overall effect of user fees on attendances and diagnoses – that which may be expected should user fees be implemented at all centres – we were unable to determine if patients were travelling to other healthcare centres outside of the district. If patients were seeking care elsewhere, then our results may underestimate the potential impact of imposing user fees at all centres. Given that the results were qualitatively highly similar when the control group was varied, the results suggest that patients were not using other centres within the district in any significant number, however further research is required to understand individuals’ decision making processes in the face of user fees when it comes to seeking care. We are also unable to determine whether the increases in cases observed after the removal of user fees was in fact partly a ‘rebound’ effect such that the increase reflects cases who waited until user fees were removed in July 2015. However, it is unlikely that pent-up demand persisted for 2 years and that the effect was reflected in acute diseases. Long term follow up is required to see whether outpatient attendances remain at the level observed after the removal of user fees, and a new free facility is opening in early 2016 in Neno District, geographically situated between two user fee facilities.

## Conclusions

Intensive efforts have been made worldwide to tackle devastating diseases such as malaria, HIV, and tuberculosis [25]. However, if patients face a barrier to access healthcare and cases are not identified then the burden of these infectious diseases will remain high. Affordability is a key ingredient in achieving universal health coverage, and we show how user fees present a barrier to healthcare in Malawi. Thus their removal may present an effective means of tackling serious diseases and a step toward universal health coverage.

## Additional file

Additional file 1: Methods - Further details about the methods are detailed here. **Table A1.** - shows results from fixed effects Poisson model: estimated effect of the presence of user fees on monthly attendances and diagnoses at health centres in Neno District, Malawi. **Table A2.** - shows results from fixed effects model: estimated effect of the presence of user fees on monthly attendances and diagnoses at health centres in Neno District, Malawi. (DOCX 20 kb)

## Abbreviations

CHAM: Christian Health Association of Malawi
MOH: Ministry of Health
PIH: Partners In Health
