# Erratum to: The impact of user fees on health services utilization and infectious disease diagnoses in Neno District, Malawi: a longitudinal, quasi-experimental study

**DOI:** 10.1186/s12913-016-1897-1

**Published:** 2016-11-04

**Authors:** S. I. Watson, E. B. Wroe, E. L. Dunbar, J. Mukherjee, S. B. Squire, L. Nazimera, L. Dullie, R. J. Lilford

**Affiliations:** 1 University of Warwick, Coventry, UK; 2 Partners In Health/Abwenzi Pa Za Umoyo, PO Box 56, Neno, Malawi; 3 Brigham & Women’s Hospital, Boston, USA; 4 Partners In Health, Boston, MA USA; 5 Liverpool School of Tropical Medicine, Liverpool, UK; 6 Ministry of Health, Neno District, Malawi

## Erratum

Upon publication of this article [1], it was brought to our attention that Fig. 1 was incorrectly presented. The correct Fig. 1 is shown below:

**Fig. 1:**
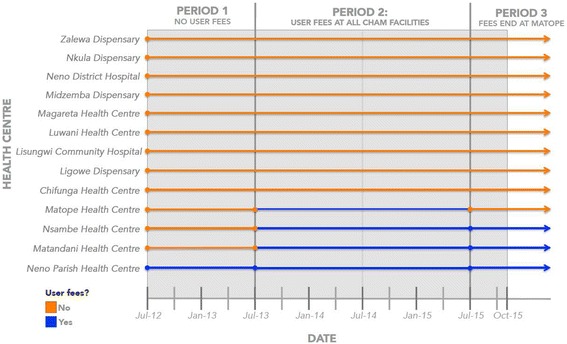
Implementation of user fees across health centres in Neno District, Malawi
