# Supplementary material for: The impact of user fees on health services utilization and infectious disease diagnoses in Neno District, Malawi: a longitudinal, quasi-experimental study
Source: BMC Health Serv Res. 2016 Oct 20;16:595. doi: 10.1186/s12913-016-1856-x (PMC5072299; doi:10.1186/s12913-016-1856-x)
Supplement: Additional file 1: — Methods - Further details about the methods are detailed here. Table A1. - shows results from fixed effects Poisson model: estimated effect of the presence of user fees on monthly attendances and diagnoses at health centres in Neno District, Malawi. Table A2. - shows results from fixed effects model: estimated effect of the presence of user fees on monthly attendances and diagnoses at health centres in Neno District, Malawi. (DOCX 20 kb) [file 12913_2016_1856_MOESM1_ESM.docx]

## ONLINE APPENDIX

METHODS

The aim of the study was to identify the effects of introducing user fees at health centres on the uptake of various health services. The use of routinely collected panel data enables us to take into account effects that may confound our analyses including secular trends in health care utilisation, seasonal effects, and unobserved health centre effects. User fees were introduced at four of 13 health centres in Neno, Malawi. The analysis presented here can be considered a generalisation of a difference in differences (DiD) model with multiple units in the treatment and control groups and multiple time periods.

We considered the following specification as the baseline model for centre $i=1,\ldots,13$ at time $t=1,\ldots,40$:

|  | $log(y_{it})=c_{i}+g_{i}t+\lambda_{t}+\tau w_{it}+x_{it}^{'}\beta+u_{it}$ | (1) |
| --- | --- | --- |

where $y_{it}$ is the outcome of interest, $c_{i}$ is an unobserved unit fixed effect, $g_{i}$ is a treatment group indicator (equal to one if a user fee introducing centre and zero otherwise), $\lambda_{t}$ are time fixed effects, $w_{it}$ is a binary indicator equal to one if unit $i$ has user fees at time $t$ and zero otherwise, $x_{it}$ are a set of observed unit characteristics, and $u_{it}$ is a random error term.

This model allows for “correlated random trends”. Different treatment groups may have different trends over time in health services utilisation, and these trends may be correlated with the introduction of user fees. For example, user fees may have been introduced in response to declining attendances. The term $g_{i}t$ relaxes the assumption of parallel trends, usually required for DiD analyses, and allows for arbitrary correlation between $g_{i}t$ and $c_{i}$.

First differencing (i.e. $\Delta y_{it}=y_{it}-y_{i,t-1}$) the model gives:

$$\Delta{log(y}_{it})=g_{i}+\eta_{t}+\tau\Delta w_{it}+{\Delta x}_{it}^{'}\beta+{\Delta u}_{it}$$

which can be estimated using fixed effects.^25^

## ADDITIONAL RESULTS

Table A1 Results from fixed effects Poisson model: estimated effect of the presence of user fees on monthly attendances and diagnoses at health centres in Neno District, Malawi

|  | **Total outpatient attendances** | **Total new malaria diagnoses, under 5s** | **Total new malaria diagnoses, over 5s** | **New confirmed HIV+ patient, aged 15-49** |
| --- | --- | --- | --- | --- |
| *Introduction of user fees^a^* | | | | |
| Estimated change (%) | -77 | -42 | -68 | -44 |
| 95% confidence interval | [-78, -77] | [-44, -40] | [-69, -67] | [-53, -35] |
| P-value | <0.001 | <0.001 | <0.001 | <0.001 |
| Number of centres | 13 | 13 | 13 | 13 |
| Number of months | 35 | 35 | 35 | 35 |
| *Removal of user fees^b^* | | | | |
| Estimated change (%) | 506 | 245 | 370 | -^c^ |
| 95% confidence interval | [488, 525] | [214, 278] | [340, 403] | - |
| P-value | <0.001 | <0.001 | <0.001 | - |
| Number of centres | 13 | 13 | 13 | 13 |
| Number of months | 27 | 27 | 27 | 27 |

The estimated change represents the average difference in monthly attendances or diagnoses associated with the introduction or removal of user fees.

^a^Estimated using data from the period July 2012 to June 2015

^b^Estimated using data from the period August 2013 to October 2015

^c^There were two few new confirmed HIV+ patients treated at the centre which removed user fees to analyse.

Table A2 Results from fixed effects model: estimated effect of the presence of user fees on monthly attendances and diagnoses at health centres in Neno District, Malawi

|  | **Total outpatient attendances** | **Total new malaria diagnoses, under 5s** | **Total new malaria diagnoses, over 5s** | **New confirmed HIV+ patient, aged 15-49** |
| --- | --- | --- | --- | --- |
| *Introduction of user fees^a^* | | | | |
| Estimated change (%) | -71 | -40 | -63 | -31 |
| 95% confidence interval | [-91, -6] | [-78, 49] | [-77, -40] | [-47, -12] |
| P-value | 0.040 | 0.25 | 0.001 | 0.007 |
| Number of centres | 13 | 13 | 13 | 13 |
| Number of months | 35 | 35 | 35 | 35 |
| *Removal of user fees^b^* | | | | |
| Estimated change (%) | 526 | 232 | 321 | -^c^ |
| 95% confidence interval | [434, 633] | [97, 460] | [180, 533] | - |
| P-value | <0.001 | <0.001 | <0.001 | - |
| Number of centres | 13 | 13 | 13 | 13 |
| Number of months | 27 | 27 | 27 | 27 |

The estimated change represents the average difference in monthly attendances or diagnoses associated with the introduction or removal of user fees. Standard errors were clustered at the health centre level.

^a^Estimated using data from the period July 2012 to June 2015

^b^Estimated using data from the period August 2013 to October 2015

^c^There were two few new confirmed HIV+ patients treated at the centre which removed user fees to analyse.
